# The Evolution of Cholesterol-Rich Membrane in Oxygen Adaption: The Respiratory System as a Model

**DOI:** 10.3389/fphys.2019.01340

**Published:** 2019-10-29

**Authors:** Juan Pablo Zuniga-Hertz, Hemal H. Patel

**Affiliations:** Department of Anesthesiology, VA San Diego Healthcare System, University of California, San Diego, San Diego, CA, United States

**Keywords:** lipid membrane, oxygen diffusion, caveolae, cholesterol, hypoxia adaptation

## Abstract

The increase in atmospheric oxygen levels imposed significant environmental pressure on primitive organisms concerning intracellular oxygen concentration management. Evidence suggests the rise of cholesterol, a key molecule for cellular membrane organization, as a cellular strategy to restrain free oxygen diffusion under the new environmental conditions. During evolution and the increase in organismal complexity, cholesterol played a pivotal role in the establishment of novel and more complex functions associated with lipid membranes. Of these, caveolae, cholesterol-rich membrane domains, are signaling hubs that regulate important *in situ* functions. Evolution resulted in complex respiratory systems and molecular response mechanisms that ensure responses to critical events such as hypoxia facilitated oxygen diffusion and transport in complex organisms. Caveolae have been structurally and functionally associated with respiratory systems and oxygen diffusion control through their relationship with molecular response systems like hypoxia-inducible factors (HIF), and particularly as a membrane-localized oxygen sensor, controlling oxygen diffusion balanced with cellular physiological requirements. This review will focus on membrane adaptations that contribute to regulating oxygen in living systems.

## Introduction

During the Paleoproterozoic era (2.5–1.6 billion years), the metabolic activity of oceanic cyanobacteria led to the atmospheric accumulation of oxygen in an event known as the GOE. The appearance of oxygen was critical for the evolutionary pressure to develop highly efficient bioenergetics through aerobic respiration to fuel the growth of life. Lipid bilayers present a highly complex molecular organization that confers a natural barrier to the free transit of molecules with a notable electric resistance (10^5^ Ω cm^2^) and capacitance (0.8–1.2 μF/cm^2^) an energetic barrier exists for the free flux of even small molecules and ions. However, considering the non-polar structure of molecular oxygen, a general assumption supposes that the energetic barrier for its diffusion, at least through the hydrocarbon moiety between membrane leaflets, should be reduced, but not absolute (Battino et al., 1968; Widomska et al., 2007; Galea and Brown, 2009). This less restricted flux of oxygen through membranes imposed a biochemical challenge to primitive aerobic organisms, namely oxidative stress. Biophysical solutions that led to highly packed lipid membranes to restrict oxygen flux arose as a possible adaptive strategy. Additionally, sophisticated mechanisms to sense oxygen to regulate activation of antioxidant responses [i.e., Nrf2 (Gacesa et al., 2016), HIF (Choudhry and Harris, 2018)] also evolved. Previous work has proposed sterols as a molecular adaptor for high oxygen (Galea and Brown, 2009; Brown and Galea, 2010), suggesting an ordered regulation of cellular oxygen beginning at the membrane and integrating into the molecular biology of the cell.

## Sterols in Membranes During Evolution: Oxygen as the Evolutive Driving Force in the Development of Cholesterol Synthesis Proteins and Membrane Sterols/Cholesterol Enrichment

### Oxygen Rise and Lipid Membrane Evolution

The GOE was a critical geobiological process that linked the evolution of photosynthetic organisms and the changes in the chemical composition of oceans. Five stages mark this event, the first of which resulted in a significant increase in atmospheric accumulation of cyanobacteria-derived oxygen approximately 2.45 billion years. The oxygen was deposited in the ocean as Fe_2_O_3_ until saturation, and subsequent excess resulted in atmospheric accumulation (Holland, 2006; Gacesa et al., 2016). The rise in atmospheric PO_2_ likely resulted in massive oxidation events that open the question of how living organisms handled ROS, a biochemical pressure that resulted in triggering the increased complexity of lipid membranes.

Lipid bilayers are vital cellular structures critical to the creation and development of life. Lipid membranes, in the earliest primitive forms of life, may have resulted in the prebiotic environment through spontaneous formation of single-chain fatty acids, saturated, and unsaturated fatty acids vesicle formation (Hanczyc et al., 2003) assisted by clay (montmorillonite) and capable of engulfing DNA and proteins (Apel et al., 2002). The presence of lipid membranes in a common ancestor has been proposed, with changes in its lipid composition between archaea and bacterial organisms (Lombard et al., 2012). Remarkably, the ability of complex sterols synthesis is restricted to eukaryote and archaea using the mevalonate pathway with only alternative isoprenoid biosynthesis occurring in bacteria (Lombard and Moreira, 2011). Genomic analysis suggested the rise of early eukaryotes (pre-mitochondrial) approximately 2.7 billion years and the origin of mitochondrial eukaryote at 1.8 billion years (Hedges et al., 2001). These match the Paleoproterozoic oxygenation event. Since oxygen availability is a *sine qua non* for final steps of sterols synthesis, the rise in atmospheric PO_2_ favors the conditions for sterol production. In this regard, the rise in atmospheric PO_2_ represents a bi-univocal hub since oxygen regulates the biosynthesis of molecules that will eventually contribute to controlling oxygen diffusion into the cell. However, if sterols, as previously suggested (Galea and Brown, 2009), emerged as a biophysical strategy to reduce oxygen diffusion through lipid membranes, why did isoprenoid biosynthesis pathway evolve in eukaryotes for the construction of complex sterols (i.e., cholesterol, ergosterol) but in bacteria remain in a less developed stage that allows the synthesis of cyclic isoprenoids. Two general assumptions potentially explain this. First, considering the higher volume of eukaryote cells, the presence of internal membranous compartments and mitochondria, the energy expenditure for membrane lipid biosynthesis is significantly higher compared with prokaryotes (Lynch and Marinov, 2017). However, limited evidence directly associates the time of appearance of mitochondria and plasma membrane complexity (Pittis and Gabaldon, 2016). Second, bacterial isoprenoid biosynthesis pathway produces hopanoids like diplopterol, sterols that can induce liquid-ordered phase formation in artificial membranes (Saenz et al., 2012), interact with phospholipids (lipid A) and determine the outer membrane order parameter of *Methylobacterium extorquens* (Saenz et al., 2015), suggesting that bacterial membrane organization and eventually resistance to free oxygen flux may not depend on oxygen availability for the synthesis of complex sterols.

The presence of sterols in lipid membranes provides unique biophysical properties that orchestrate functional events relevant to cell physiology. Therefore, the emergence of cholesterol as an evolutionary consequence to restrict oxygen diffusion, resulted in a versatile strategy to functionally organize the membrane, completely modifying cell biology leading to more sophisticated mechanisms to deal with oxygen transport through the membranes, so as elaborate response strategies to deal with changes in oxygen availability, like hypoxia events.

### Membrane Permeability

Phospholipids, as building blocks of membranes, result in notable effects on biophysical properties (e.g., structural changes in polar headgroups, hydrocarbon chains, and the proportion of specific phospholipids) to directly impact biological and physiological processes. The presence of a highly hydrophobic core and, depending on the specific composition of polar head groups, lipid membranes are virtually impermeable providing a distinct electrochemical and biochemical composition between extracellular and intracellular compartments. This barrier acts as a capacitor to influence a wide range of processes (Ray et al., 2016). When considering the permeability to non-polar molecules (gases like CO_2_ and O_2_), a general inspection may suggest a less restricted permeation through the membrane, with tremendous implications in gas exchange and molecular organization of respiratory systems.

When considering the flux of a substance (*J*) through lipid membranes, there are two elements to consider: a concentration gradient Δ*C* (chemical/electrochemical gradient) and the membrane permeability coefficient *P*_M_. Membrane permeability is a more complex concept that involves other parameters such as the partition coefficient, the diffusion of the molecule through the membrane, and the resistance. These terms are mathematically integrated into an expression valid for non-electrolyte flux through the *z* plane of the lipid membranes (Diamond and Katz, 1974):

1/P=M-(ΔC)/J=r′+∫dx/K(z)D(z)+r″

with *K*(*z*) as partition coefficient, *D*(*z*) as diffusion coefficient, and *r* resistance of the head groups. In molecular terms, the concentration gradient through the membrane represents the driving force that allows the flux of ions through membranes. Membrane resistance, partition coefficient, and diffusion are variables that depend directly on the membrane lipid (and protein) composition. The diffusional coefficient, time that a molecule takes to pass through an area, is normally expressed for gases as apparent diffusion (Fischkoff and Vanderkooi, 1975; Denicola et al., 1996; Moller et al., 2005) due to technical artifacts and the difficulty to measure the real concentration (or partition of the substance in the membrane) due to complex composition and consequent behavior of lipid membranes. In this regard, diffusion is related to the partition coefficient, the ratio of the concentration of a substance in the membrane and buffer/extracellular media, that is directly related to the solubility of a substance in the membrane and depends on its chemical nature, charge, size and in lipid composition, sterols concentration, temperature, phase transition and membrane homogeneity of the membrane (Windrem and Plachy, 1980; Raguz et al., 2009). This last factor is critical since phospholipids present three-dimensional organization depending on the polar head group and acyl chain compositions, resulting in varied order parameters and domain arrangements throughout the cell membrane.

Several states of arrangement can be recognized (Van Meer et al., 2008), like liquid-ordered domains (*L*_o_) or regions with high molecular order and fewer degrees of freedom for lipid rotation and phase transition, and liquid disordered phase (*L*_d_), with opposite motion and freedom degree characteristics. As can be expected, the partition of substances like gases in both membrane phases can differ dramatically, which is also influenced by the membrane molecular order degree. In this regard, lipid membranes without cholesterol present low resistance for the permeation of non-polar molecules like oxygen. On the other hand, the higher the cholesterol content leading to membrane organization complexity, the bigger the membrane resistance to oxygen permeation (Widomska et al., 2007; Mainali et al., 2013). In the present work, we will focus mainly on the effect of cholesterol in membrane biophysics and the impact on oxygen diffusion.

### Oxygen Diffusion Through Lipid Membranes

For terrestrial aerobic organisms, oxygen participates in a multitude of reactions that propitiate life. Many studies have been performed regarding oxygen gas exchange, transport, and ultimately, its involvement in biochemical and physiological reactions as oxygen is critical for life and a significant factor in organismal and organ physiology and pathophysiology. However, oxygen diffusion through lipid membranes, the ultimate barrier from alveolar cells to red blood cells and peripheral tissues, is not entirely understood. Oxygen, as a non-electrolyte, may permeate the plasma membrane through lipid bilayer leaflets. Membrane permeability to small molecular size solutes has been thoroughly studied, leading to the proposal of several mechanisms. Of these, the solubility-diffusion model (Volkov et al., 1997; Shinoda, 2016), or Overton’s law (Overton, 1896), associates permeability with the partition coefficient of the solute and its diffusion. Consequently, the solute permeability relates to membrane properties. In living systems, the membrane lipid composition and its molecular organization are critical parameters that will determine oxygen flux.

Membrane diffusion of small solutes according to Overton’s law depend on membrane permeability and ultimately in its biophysical properties, suggesting that modifications in membrane structure may increase the permeability of such solutes. Indeed, Thomae et al. (2007) show controversial data proposing that above a critical cholesterol concentration in PC liposomes, there is no effect in the permeation of aromatic molecules like sialic acid. Missner et al. (2008) showed that adding cholesterol to planar PC lipid bilayers (PC:Chol = 1:1) indeed decreases sialic acid permeability, suggesting the importance of cholesterol in permeability, as Fick’s law and Overton’s law propose. Oxygen permeability has been shown to be in the order of 210–230 cm/s in DMPC bilayers, consistent with Overton’s law (Dzikovski et al., 2003), in the absence of cholesterol at 37°C, suggesting that oxygen diffusion through lipid membranes is a fast process, which may have been the case for primitive organisms. However, the importance of the raise of sterols in membrane evolution in complex organisms may have been a means to increase membrane complexity and with it limit oxygen diffusion to better control biological reactions (i.e., mitochondrial electron transport). In fact, the energetic barrier for oxygen diffusion in POPE and POPC membranes is smaller than transporter-mediated AQP1 oxygen diffusion (Hub and De Groot, 2008). Notwithstanding, this rule does not take into account structural inhomogeneities in cellular membranes (Missner and Pohl, 2009) rich in cholesterol such as lipid rafts (Hannesschlaeger et al., 2019) and caveolae, suggesting that the application of Overton’s law to membranes of complex organisms is still controversial. In this regard, we will focus this review on the influence of cholesterol and cholesterol-rich domains such as caveolae on oxygen diffusion through lipid membranes in biological systems.

**FIGURE 1:**
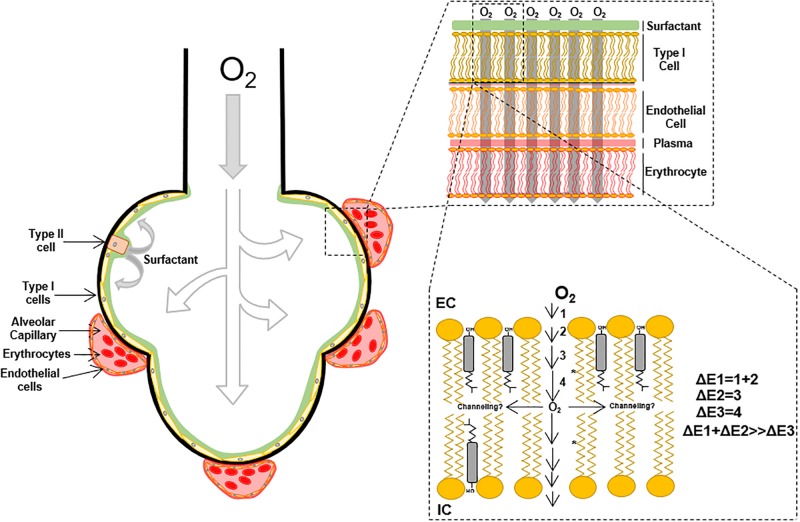
Overview of molecular oxygen flux in the lipid bilayer.

Small molecules permeability across the lipid membrane is related to the partition coefficient (Shinoda, 2016) in the membranes moieties. According to the chemical properties of the solute, stabilization will be achieved in solution by the solvent or complex formation with other molecules. When considering oxygen flux through membranes, the first step is the transfer from the solvation moiety to the polar head groups and subsequent entry into the hydrocarbon moiety subregions. These transfer steps represent energetic barriers, ΔE, that oppose or favor oxygen flux according to the chemical properties; ultimately, the sum of all ΔE’s determine oxygen flux through the membrane (Figure 1), in other words, the total membrane resistance (Moller et al., 2016). Because of oxygen polarity, a lower energetic barrier for the permeation into the hydrocarbon acyl chain moiety is expected. However, the first portion of the membrane, until C9 is a region with high molecular order, restricting oxygen free flux. This effect is associated with lipid spatial organization that depends on temperature, phospholipid acyl chain structure (i.e., presence of saturations), the presence of sterols like cholesterol and ultimately proteins that may induce transient changes in membrane order parameter (Subczynski et al., 2012; Mainali et al., 2013). After passing through this ordered region, a considerable drop in the energetic barrier results as oxygen enters the hydrophobic core in the center of the lipid bilayer.

Many efforts have attempted to determine oxygen transport through plasma membrane using techniques such as fluorescence quenching of pyrene (Fischkoff and Vanderkooi, 1975; Denicola et al., 1996; Moller et al., 2005) and spin-label probe detection through EPR (Widomska et al., 2007; Raguz et al., 2009, 2011; Subczynski et al., 2011). In the later, the oxygen transport parameter, *W*_(_*_x_*_)_, representing the collision rate between the spin-label probe and oxygen molecules, accounts for oxygen transport through lipid membranes (Kusumi et al., 1982; Widomska et al., 2007). Employing *W*_(_*_x_*_)_^–1^, a measurement of the membrane resistance to oxygen permeation, Widomska et al. (2007) showed increases in membrane resistance to oxygen permeation in different artificial membranes preparations composed of POPC, cholesterol and calf lipid lens (POPC < POPC/Cholesterol < calf lipid lens). These studies suggest that oxygen transfer to the phospholipid headgroups and diffusion to C9 is the most energetically restricted. Consequently, the partition coefficient [*K*(*x*)] and diffusion [*D*(*x*)] changes across the lipid bilayer (Subczynski et al., 2017; Plesnar et al., 2018), make it difficult to determine the actual diffusion of oxygen through lipid membranes. Moller et al. (2016), determined the oxygen corrected diffusion coefficient in multilamellar vesicles defined as the apparent diffusion corrected by the oxygen partition coefficient. *K*(*z*) is related to the partial molar volume of the phospholipids and their free volume that is directly associated with temperature and unsaturation in the acyl carbon chains of the phospholipids (Smotkin et al., 1991; Widomska et al., 2007; Moller et al., 2016). Consequently, the higher the temperature and the number of unsaturated bonds, the higher the free volume of the phospholipid, leading to free volume pockets in the membrane that can accommodate oxygen molecules. Interestingly, the presence of cholesterol in artificial membrane preparations increases membrane resistance to oxygen permeation, which can be partly explained by a reduction in the membrane-free volume (Subczynski et al., 1989; Falck et al., 2004; Widomska et al., 2007; Angles et al., 2017). Recent simulation analysis determined oxygen diffusion time depending on membrane composition (De Vos et al., 2018a, b), confirming the impact of acyl chain unsaturation.

In addition to oxygen diffusion through lipid membranes, channel-mediated oxygen diffusion can occur. Human erythrocytes aquaporins inhibition resulted in a reduction in oxygen diffusion through deoxyhemoglobin-oxyhemoglobin differences (Ivanov et al., 2007), suggesting aquaporins as an alternate mechanism for oxygen diffusion. Simulation studies suggest AQP1 is permeable to gases (Wang et al., 2007, 2010). Overexpression of AQP1 in PC12 cells resulted in increased oxygen diffusion, and rats exposed to hypoxia showed increased lung AQP1 gene expression (Echevarría et al., 2007), expression possibly regulate by HIFs. Hypoxia (10% oxygen) also induced AQP1 expression in mouse lung and brain (Abreu-Rodriguez et al., 2011). *In vitro* studies showed hypoxia-induced AQP1 expression, and that the same expression links to hypoxia inducible factor 1α (HIF-1α) (Abreu-Rodriguez et al., 2011; Tanaka et al., 2011). Mice chronically exposed to hypoxia (10% oxygen) showed increased AQP1 expression in lung tissue resulting in vascular remodeling (Schuoler et al., 2017). In this regard, the physiological implications of this alternative pathway for oxygen diffusion suggest that AQP1 may facilitate oxygen diffusion in particular membranes (Clanton et al., 2013). Thus, AQP1 expression in erythrocytes may not only be associated with osmotic control (Huang Y.C. et al., 2019), but also oxygen diffusion. AQP1 may be a means in red blood cells to overcome the limitation imposed by the high membrane cholesterol content (50 mol%) (Steinbach et al., 1974; Menchaca et al., 1998; Buchwald et al., 2000a; Dotson et al., 2017). AQP1 expression is related to high oxygen [and other gases like CO_2_ (Blank and Ehmke, 2003)] diffusion especially in high demand tissues like lung, carotid body, erythrocytes and microvasculature endothelial cells (Borok and Verkman, 2002; Echevarría et al., 2007; Munoz-Cabello et al., 2010; Abreu-Rodriguez et al., 2011; Crisp et al., 2016; Huang Y.C. et al., 2019). However, data from AQP1 knockout mice suggest a minimal role of aquaporins in oxygen diffusion (Al-Samir et al., 2016), in line with previous dynamic simulation studies that suggest poor permeation of hydrophobic molecules like oxygen and CO_2_ through AQP1 (Hub and De Groot, 2008).

### Cholesterol Decreases Free Volume Pockets in Membranes Reducing the Oxygen Diffusion/Partition Coefficient

The biophysical effects of cholesterol on membrane structure and properties have been extensively studied in artificial and cell-derived membrane preparations, as well as *in silico* simulations. Cholesterol content in cellular membranes modulates a broad range of phenomena, from controlling light path in eye lens (Widomska et al., 2007; Angles et al., 2017; Subczynski et al., 2017) to oxygen flux control in red blood cells (Kon et al., 1980; Buchwald et al., 2000b; Luneva et al., 2007); however, its influence in solute permeation and diffusion is less well understood. The increase in membrane resistance to oxygen permeation due to the increase in lipid composition complexity and cholesterol concentration (Widomska et al., 2007), is a consequence of sterols effect on membrane molecular/biophysical parameters by restricting phospholipid freedom degrees (Ipsen et al., 1989; Khelashvili and Harries, 2013), free volume (Marrink et al., 1996), and membrane order (enthalpy) parameters (Halstenberg et al., 1998). Of these, the free volume is a critical factor in the diffusion of small solutes through lipid membranes.

Simulation analysis in DPPC systems showed non-homogeneous distribution of free volume in the lipid membrane, with lower empty volume in phospholipid headgroups, due to electrostatics and bond hydrogen attractions, and in the highly ordered region of acyl carbon chains [previously predicted by EPR as being the first 9–10 carbons (Marrink et al., 1996; Widomska et al., 2007; Subczynski et al., 2012)]. With a molecular diameter near 0.3 nm, oxygen can occupy 10% of the free volume in the center of lipid bilayers, reducing to less than 2% in the condensed ordered region of acyl chains. In this regard, variations of *K*(*z*) and *D*(*z*) across the lipid membrane may be due, partly, by changes in free volume. The addition of cholesterol up to 50 mol% in single phospholipid membrane simulations showed an increase in the free energy required to transfer small solutes from water environment/polar head groups to the lipid acyl chains, directly affecting partition coefficient (Wennberg et al., 2012).

Phospholipid acyl chain composition also influences oxygen diffusion. DPPC membrane systems present higher oxygen diffusion than unsaturated phospholipids POPC and DOPC (De Vos et al., 2018a). Cholesterol insertion in lipid membranes is stabilized by Van der Waals’ interaction with acyl carbon chains (Wennberg et al., 2012), creating ordered arrangements that reduce the partition coefficient of small molecules (Zocher et al., 2013) and restricted phospholipid acyl chains *trans*-gauche rotation, leading to increased membrane rigidity (Cassera et al., 2002; Molugu and Brown, 2019). These factors decrease membrane-free volume and free pockets that may accommodate oxygen, reducing its partition and diffusion.

Therefore, the oxygen molecules incorporated in the membrane will preferentially distribute in the center of the bilayer, where the free volume is higher. EPR and simulation data suggest that cholesterol incorporation in POPC membranes leads to a 10% reduction in oxygen permeability. However, oxygen solubility (and diffusion) is higher in the center of the membrane (Dotson et al., 2017), suggesting the possibility of oxygen channeling in the center of the lipid bilayer (Figure 1). In addition to the cholesterol effect in membrane oxygen permeability, the insertion of proteins into lipid bilayers negatively contributes to oxygen permeability (Ashikawa et al., 1994). Atomistic simulations suggest more than 40% reduction in oxygen permeability after the incorporation of ungated K^+^ channels (Dotson and Pias, 2018). Altogether, oxygen diffusion through lipid membranes is a process that can be restricted by the increase in the lipid bilayer complexity, through sterols, specific phospholipid composition, and proteins incorporation.

## Oxygen-Dependent Cholesterol Distribution in Membranes: From Lungs to Red Blood Cells and Tissue Oxygenation

### Cholesterol as a Cornerstone for Oxygen Adaption, and Its Relation to Cell Physiology

**FIGURE 2:**
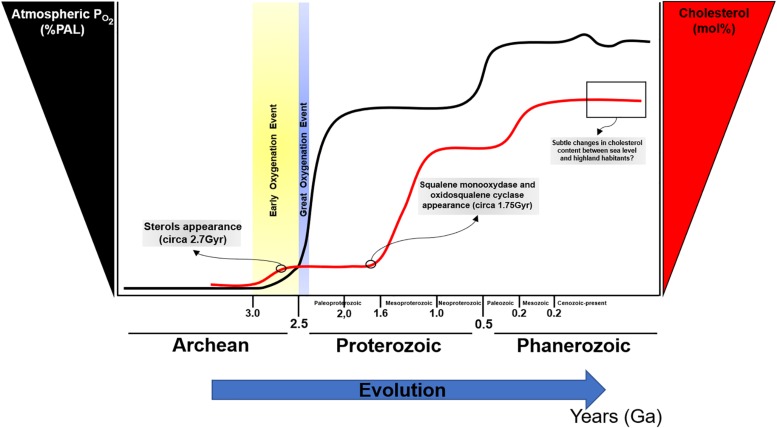
Oxygen and cholesterol changes during evolution. PAL, present atmospheric level. Modified from [opetwcite]B96,B107[clotwcite]Kump (2008); Lyons et al. (2014), and Gold et al. (2017).

The rise in atmospheric oxygen 2.32 billion years ago (Bekker et al., 2004) led to significant changes in life. Fossil evidence suggests the appearance of sterols after the GOE, approximately 2.7 billion years ago (Brocks et al., 1999; French et al., 2015), presumably as an evolutionary strategy to reduce oxygen diffusion; paradoxically, sterols synthesis appears to be highly dependent on oxygen for its biosynthesis (Debose-Boyd, 2008). Interestingly, phylogenetic and molecular clock analyses of two sterol biosynthesis enzymes suggest that squalene monooxygenase and oxidosqualene cyclase appeared previously, approximately 1.75 billion years ago (Gold et al., 2017). It is possible that after an atmospheric increase in PO_2_, sterols biosynthesis was co-opted to rapidly evolve diverse cellular processes as a strategy to maintain optimal oxygen levels for productive growth and maintenance of biological functions (Figure 2). With the increase in complexity of sterols, particularly in eukaryotes, cholesterol biosynthesis pathways became intertwined with a wide range of molecular events [i.e., insulin secretion and beta-cell physiology (Tsuchiya et al., 2010; Zuniga-Hertz et al., 2015; Veluthakal et al., 2016; Syeda and Kowluru, 2017), intracellular pathway regulation by isoprenylation (Yang et al., 2015; Zhang X. et al., 2018), brain and neuronal function (Sun et al., 2015; Moutinho et al., 2016, 2017; Berghoff et al., 2017; Ferris et al., 2017; Paul et al., 2018), cardiac and myocytes physiology (Haque et al., 2016; Gadeberg et al., 2017; Hissa et al., 2017; Russell et al., 2017), cellular membrane-associated events (Kreutzberger et al., 2015; Stratton et al., 2016; Yang et al., 2016; Guixa-Gonzalez et al., 2017)]. In this sense, cholesterol can be considered as a molecular hub and a major control point for the rise and evolution of life as it currently exists.

Cholesterol biosynthesis is high energy and oxygen demanding process (Debose-Boyd, 2008). The balance between the requirement of oxygen for the synthesis of a molecule that ultimately regulates oxygen diffusion may represent a paradox. Since key enzymes for sterols biosynthesis appeared before the GOE, the original notion that sterols appeared in evolution to counteract the increase in atmospheric PO_2_ can be ruled out. Notwithstanding, the complex configuration of cholesterol as a critical molecule for diverse biochemical pathways regulating feedback control loops, may suggest the cellular adaption to maintain a constant cholesterol level to ensure membrane integrity to avoid hazardous levels of oxygen diffusion. Interestingly, cholesterol presence in lipid membranes is a crucial molecule that led to phase separation allowing for the formation of membrane nanodomains such as lipid rafts.

### Cholesterol Distribution in Cell Types: From Lungs to Red Blood Cells

With the emergence of terrestrial organisms, more complex respiratory systems and gas exchange mechanisms emerged. The increase in bioenergetic metabolism complexity and its reliance on oxygen led to complex organisms developing sophisticated mechanisms for gas exchange and transport. The presence of cholesterol as a pivotal molecule for membrane organization in the respiratory systems has been documented, leading to profound biophysical, functional, and physiological implications. Cholesterol concentration in specific cell systems suggests a non-random distribution. The lens fiber of the eye and alveolar epithelia, both of which have direct contact air, show high densities of cholesterol (Ray et al., 2016) with the environment. The membranes of lens fiber cells have been extensively studied to understand oxygen diffusion, because of the particularly high cholesterol content that ranges from 2 (lens cortex) to 4 (lens nucleus) cholesterol/phospholipid molar ratio (Li et al., 1987; Widomska et al., 2007, 2017). These levels can be impacted by age (Mainali et al., 2017) and may underpin the development of cataracts due to increased oxidation. Lung epithelial cells, particularly type I cells, even though their exposure to air is not direct also present an essential dependence in membrane cholesterol for their role as the primary conduit for oxygen diffusion.

The human lung alveoli, the main structures for gas exchange, present differential cholesterol function at each level of oxygen movement from the air into the body (i.e., surfactant and respiratory epithelium composed of type I and type II pneumocytes (hereafter named as type I and II cells), alveolar capillary endothelial cells, erythrocytes, and ultimately cells of end organs were oxygen is delivered). Pulmonary surfactant, secreted by type II pneumocytes, is a mixture of proteins (10%) and lipids (90%) composed of phospholipids (DPPC, PC) and to a lesser extent, cholesterol (5–10%) (Zuo et al., 2008; Bernhard, 2016). It constitutes the first membrane barrier between the air and respiratory system, preventing alveoli collapse during expiration by reducing surface tension. The role of cholesterol here is not entirely clear. Cholesterol depletion in porcine surfactant negatively affected membrane molecular arrangement increasing membrane phase segregation (Andersson et al., 2017). Simulations in artificial preparations suggested that cholesterol associated with the SP-C impacted membrane organization dependent on temperature (Roldan et al., 2017). This evidence may give clues as to the physiological adaptions to temperature. Changes in temperature may lead to subtle changes in membrane Tm impacting molecular arrangements.

Different species show different concentrations in surfactant and cholesterol, with higher cholesterol:phospholipid proportion in reptiles and other species, likely an adaption to maintain constant surfactant fluidity upon changes in body temperature (Orgeig and Daniels, 2001). Changes in cholesterol concentration may alter surfactant lipid ordering arrangements, directly affecting surface tension and other mechanical properties. Due to the surfactant membrane composition being enriched in saturated acyl chain phospholipids, alterations in cholesterol concentration will modulate order parameters and ultimately molar free volume, with direct consequences in oxygen diffusion. This ratio is particularly crucial for adaption mechanisms to hypoxia, high altitude, and clinical conditions related to reduced lung diffusive capacity.

The alveolar surface is covered by type I and type II cells, constituting the first cellular barrier for gas exchange, which is facilitated by cell flattening during inspiration, as well as by the PO_2_ gradient. The plasma membrane is the ultimate barrier for oxygen diffusion from alveoli to capillary endothelium. Lipid homeostasis plays a crucial role in alveolar physiology, particularly for type I cell membrane composition and for the control of type II cell-derived surfactant lipidic composition. ABCA3 and ABCG1 transporters are involved in surfactant composition and respiratory failure associated with mutations and deficiencies in these transporters (Bates et al., 2008; Plantier et al., 2012; De Aguiar Vallim et al., 2017; Tang et al., 2017). Type I cells cover 95–98% of the alveolar surface (Stone et al., 1992); present a planar morphology, and are 100 times thinner than the cuboidal type II cells (Jansing et al., 2017). Interestingly, studies performed in the early 1980s’ show the presence of cholesterol-rich nanodomains, caveolae, in rabbit type I cells membrane (Gil et al., 1981), indirectly suggesting the presence of cholesterol in higher concentrations compared to surfactant, as a potential explanation of reduced oxygen diffusion rate. However, during inspiration, the alveolar PO_2_ (100 mmHg) > capillary PO_2_ (40 mmHg) difference works as a driving force for oxygen diffusion in a process that takes a fraction of a second (red blood cell transit through alveolar capillaries: 0.25–0.75 s) (Petersson and Glenny, 2014), suggesting as well that type I cell membranes may present a molecular organization that facilitates rapid gas diffusion.

Oxygen diffusion from alveolae to erythrocytes depends on the resistance offered by membrane barriers, as well as the diffusion into erythrocytes. DMO_2_, depends on a tissue to blood plasma barrier. The tissue barrier is composed of type I cells and capillary endothelial cells. DMO_2_ is defined by an equation that integrates a permeation coefficient (Krogh’s coefficient, which integrates tissue-membrane diffusion and oxygen solubility, 3.3 × 10^–8^ cm^2^/min × mmHg), surface area, and barrier thickness (Roughton and Forster, 1957; Hsia et al., 2016). Despite the resistance that type I and endothelial cell plasma membranes may oppose to oxygen diffusion, this parameter is minimized by the anatomical alveolar organization with flattened type I cells, minimal distance to the endothelial cell, and surface area that covers 95% of the alveolae.

Thus, oxygen flux from alveolae to erythrocytes is directly associated with transmembrane diffusion, a process that depends on lipid bilayer order parameters. Oxygen diffusion is associated with cholesterol; it follows a specific pattern of its concentration from the surfactant, type I cells/capillary endothelial cells, and onto erythrocytes membranes. The functional association of the cholesterol content of erythrocytes has been extensively studied. Erythrocytes present a particularly high membrane cholesterol content, with a cholesterol: phospholipid ratio of 1:1. A negative correlation exists between blood oxygen diffusion and total plasma and erythrocytes membrane cholesterol concentration (Buchwald et al., 2000b). Reduction in total plasma cholesterol by 12 weeks simvastatin treatment reduces erythrocyte membrane cholesterol (33%) with a significant increase in blood oxygen diffusion rate (Menchaca et al., 2004).

Additionally, rabbits supplemented with cholesterol showed increased erythrocyte cholesterol content and reduced oxygen diffusion (Menchaca et al., 1998). Cholesterol molecular/spatial organization in time stable submicrometric domains in erythrocyte lipid membrane (Carquin et al., 2015) suggested singularities in lipid bilayer organization associated with oxygen diffusion and transport; the characteristic cellular shape of erythrocytes associated with increased gas exchange capacity. Recent data suggest cholesterol-enriched membrane domains distribute within high curvature areas of erythrocytes membrane; MβCD, cholesterol depletion did not affect cell shape but impaired cellular deformability (Léonard et al., 2017), that is essential for erythrocyte flux through microvasculature.

Notwithstanding, the enrichment in membrane cholesterol content still leaves open the question of the membrane rigidity increase in erythrocytes, and the suggested reduction in oxygen diffusion (Dumas et al., 1997), affecting lung diffusing capacity. Theoretical studies that combined mathematical analysis and observed physiological parameters suggest an oxygen lung diffusion capacity of 158 mL O_2_/ml mmHg. Resistance to oxygen diffusion is a multifactorial value composed of the alveolae and capillary membrane (i.e., type I cells and endothelia), plasma, oxygen binding with hemoglobin and erythrocyte diffusion. The smaller capillary diameter and, the lower plasma-derived resistance due to a reduction in its volume are conditions in which membranes are mainly responsible for diffusion resistance. These simulations suggest that resistance to oxygen diffusion presents a more significant contribution of the plasma, erythrocyte, and membrane components (Roy and Secomb, 2014). It can be hypothesized that, even considering the less free volume in erythrocyte membrane hydrophobic domain because of high cholesterol concentration may reduce oxygen diffusion and partition in lipid bilayer, this is overcome by whole-cell morphology adaption that increases surface and oxygen exchange (Hsia et al., 2016), pulmonary capillary diameter modification, and alveolar/erythrocyte PO_2_ differences. Under conditions that affect alveolar epithelial and membrane structure, higher resistance to gas diffusion with consequent lower blood oxygen saturation is expected. However, at the level of peripheral tissues that are supplied with oxygen, with different rates of oxygen consumption, more sophisticated mechanisms are required to provide additional control mechanisms (i.e., hypoxia, adaption to extremes, and exercise).

### Caveolae as Oxygen Sensors

A growing body of evidence suggests the presence of cholesterol-rich caveolae domains in lipid membranes of alveolar epithelia (Gumbleton, 2001; Williams, 2003; Jung et al., 2012), providing an essential modulatory function critical during hypoxia (Botto et al., 2006). Caveolins, 17–24 kDa proteins required for caveolae formation, regulate several biological processes associated with the plasma membrane, important in physiology (Busija et al., 2017). All isoforms of Cav1-3, have been detected in lung, with greater extent of Cav1-2 in alveolar epithelial cells (in type I and type II cells, respectively) and capillary endothelium. Cav1 is critical for reducing membrane tension induced by hypotonic stress; Cav1^–/–^ or WT MLEC treated with MβCD presented an increase in membrane tension (Sinha et al., 2011). Caveolae are important molecular platforms to regulate cellular function; alveolar epithelia caveolae respond to functional modulation. Early studies in Cav1 null mice reported the loss of caveolae in alveolar epithelial cells and alveolar capillary endothelial cells accompanied with hyperactivation of NOS with no alterations in lipid rafts protein profile and lipid composition (Drab et al., 2001). Numerous studies have shown an abundance of caveolae in alveolar epithelial and pulmonary endothelial cells (Newman et al., 1999; Barar et al., 2007); chronic hypoxia-induced pulmonary hypertension reduced epithelial cell membrane cholesterol and inward Ca^2+^ flux, however, caveolae integrity was maintained (Paffett et al., 2011). On the contrary, morphological and functional evidence has shown the absence of caveolae in type II cells (Fuchs et al., 2003; Ikehata et al., 2009; Jung et al., 2012). In this regard, the presence of caveolae in oxygen diffusion specialized type I cells and the absence in surfactant-secreting type II cells, suggest a functional relation of caveolae as oxygen sensors and transporters. Other functions have been proposed regarding the increased surface area that caveolae create in type I cell membranes, including protein transcytosis and localization of Na^+^ pump (Maniatis et al., 2012) and expression and localization of aquaporins (Dobbs et al., 1998; Gao et al., 2015; Hou et al., 2015).

Lung capillary endothelial cells depend extensively on caveolae and Cav1-mediated cell signaling that regulates cellular adaptions promoting gas diffusion. Numerous studies have shown a structural and functional association of eNOS with caveolae and Cav1 (Maniatis et al., 2006; Bernatchez et al., 2011; Chen et al., 2012; Trane et al., 2014). The negative regulation of eNOS by Cav1 constitutes a fine-tuning mechanism to control oxygen diffusion into erythrocytes. The Cav1 scaffolding domain presents a binding subdomain (residues 90–99) that interacts with eNOS with *Kd* 42 nM; when a mutation in residue 92 (F92A) which regulates eNOS is introduced (Bernatchez et al., 2011), eNOS-derived NO production is induced (Trane et al., 2014), with a concomitant capillary dilation. Paradoxically, the increase in capillary diameter increases the plasma volume, opposing a higher resistance to oxygen diffusion capacity (Roy and Secomb, 2014), which probably is surpassed by the higher amount of erythrocytes flux, giving a resulting net increase in oxygen diffusion. In this sense, caveolae behave as an oxygen sensor to regulate molecular and physiological adaptions, promoting erythrocyte-mediated blood oxygen transport. The molecular mechanism that triggers Cav1 release of eNOS, how it is related to oxygen as a trigger, and how changes in oxygen diffusion transduces into caveolae modifications that promote eNOS release are not entirely understood.

**FIGURE 3:**
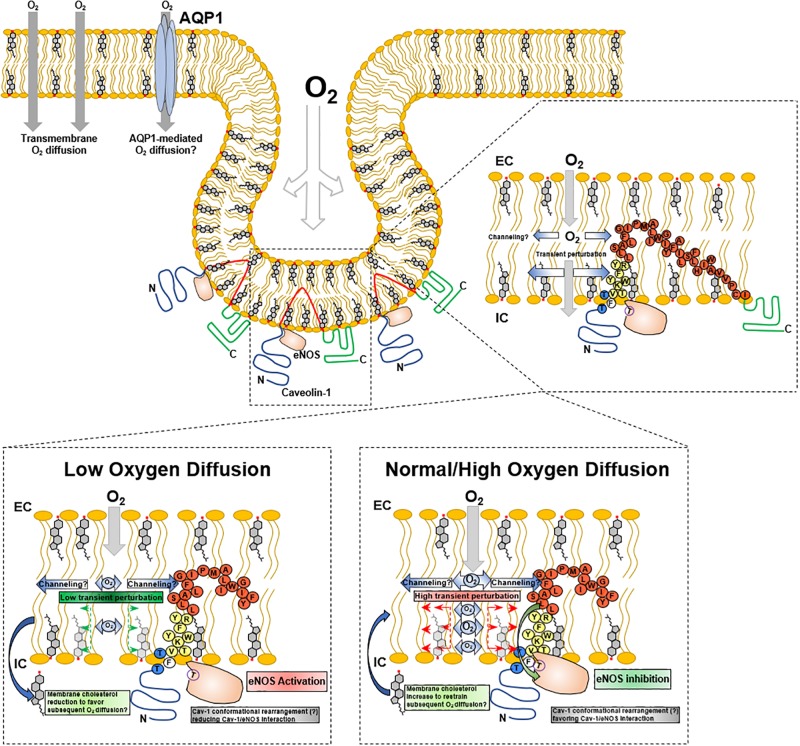
Cav1 mechanotransduction hypothesis upon O_2_ membrane diffusion. Inset, adaption from Busija et al. (2017).

In the context of alveolar-capillary endothelia, it can be hypothesized that Cav1 may detect oscillations directly or indirectly related to alveolar-endothelial oxygen diffusion and induce conformational changes leading to release eNOS inhibition, with the consequent increase in NO, alveolar-capillary dilation and erythrocytes gas exchange. This possible mechanism may imply a mechanosensory behavior of Cav1. When oxygen diffuses through lipid membranes, the result may be a transient molecular organization perturbation within polar head groups and proximal hydrophobic moiety (acyl chain carbon 9–10) that are detected by Cav1 scaffolding and membrane domains, conducting conformational structure modifications that promote/facilitate Cav1-eNOS binding through π-π interactions between Phe92 and eNOS Trp447 (Trane et al., 2014). Subtle reductions in oxygen diffusion will reduce oxygen-derived membrane perturbation, leading to Cav1 modifications that will “loosen” Cav1-eNOS interaction, promoting the activation and release of NO. Consequently, Cav1 may act as an oxygen sensor through transient membrane mechanical transduction (Figure 3).

Caveolae are associated with mechanotransduction. *In vitro* stimulation of endothelial cells with volume flow, shear stress induces increased membrane caveolae distribution and acute changes in shear stress increased Cav-1 and eNOS phosphorylation (Rizzo et al., 2003). Cav-1 KO mice present impaired flow-induced arterial dilation due to eNOS-derived NO (Albinsson et al., 2007). *In vitro* ischemia in lung endothelial cells showed a modulatory role of ROS and phosphorylation of ERK1/2, leading to eNOS activation and NO synthesis; membrane disruption by cyclodextrins inhibited ERK1/2 phosphorylation, suggesting the involvement cholesterol-rich membrane domains (Wei et al., 2001). Similar studies showed the involvement of Cav1 as a shear flow sensor in endothelial cells (Milovanova et al., 2008), and the involvement of PECAM1 (Noel et al., 2013). Caveolae are proposed to behave as a buffer for mechanical forces in pulmonary arterial smooth muscle cells, modulating the activity of stretch-activated Ca^2+^ channels (Gilbert et al., 2016). The dynamic structure of caveolae also flattens in response to cellular membrane stretching (Parton and Del Pozo, 2013; Shihata et al., 2016), suggesting not only a protective role but also a functional adaption. Such physical changes in caveolae may be a means to increase cell surface area and reduce volume to facilitate oxygen diffusion in alveolar capillaries. The necessity of increased oxygen supply to a particular tissue, demands a local control mechanism that will adjust the conditions to increase oxygen provision. In the same way, peripheral tissues capillary endothelia may behave in the same way as in alveolar capillary. Through changes in local oxygenation conditions, the caveolae mechanosensory system may activate signaling pathways that will activate eNOS and other molecular adaptors to regulate oxygen diffusion.

Little evidence suggests a functional role of caveolae and caveolins in erythrocytes. The formation of lipid rafts is described in erythrocyte membranes and their functional role in the initiation of signaling cascades (Mikhalyov and Samsonov, 2011; Ciana et al., 2014; Biernatowska et al., 2017; Mcgraw and List, 2017). Cav-1 is associated with erythrocytes DRMs, playing a particular role during Alzheimer’s disease by binding circulating amyloid-β (Hashimoto et al., 2015). No evidence exists to support the possible functional role of caveolae/Cav-1 as an oxygen sensor in erythrocytes. Even though recent reports suggest eNOS activity in erythrocytes (Dei Zotti et al., 2018), many studies suggest no Cav-1 expression in these cells, ruling out the potential for mechanosensory regulation by Cav1 in these cells. Nevertheless, molecular mechanisms that affect erythrocyte plasma membrane fluidity and order parameter may have consequences in the modulation of oxygen diffusion. The blockade of MPP1 interaction with flotillin in human erythrocytes reduced membrane order properties, suggesting a mechanism that regulates lipid rafts formation in erythrocytes (Biernatowska et al., 2017). The molecular coupling of this mechanism with a triggering system that responds to modifications in PO_2_ or other mechanical stimuli may behave as an adaption that regulates erythrocytes membrane fluidity/rigidity modifications and cholesterol-rich domains organization, ultimately favoring or restricting oxygen diffusion. The environmental challenges that lead to changes in oxygen availability, i.e., hypoxia, demand molecular adaptions that lead to fine-tuned regulation of oxygen diffusion from the environment through lung epithelia to erythrocytes, as well as into tissues.

### Other Proteins as Oxygen Sensors and CRAC Domains

Membrane-associated proteins are non-randomly distributed. CRAC domains (Leu/Val-X_1__–__5_-Tyr-X_1__–__5_-Arg/Lys) are motifs associated with guiding protein distribution/localization into cholesterol-rich membrane domains, that experimentally can be isolated as DRMs. Other cholesterol-recognition domains like CARC domains (Lys/Arg-X_1__–__5_-Tyr/Phe-X_1__–__5_-Leu/Val) on proteins may regulate specific thermodynamic interaction with cholesterol (Di Scala et al., 2017). Cav-1 CRAC sequence (^94^VTKYWFYR^101^; Figure 3, yellow) has been proposed to be associated with the insertion into the lipid membrane (Busija et al., 2017). In this regard, if caveolae play a crucial role as an oxygen sensor platform, proteins associated with this function, other than Cav-1, may also present with cholesterol recognition domains. As previously stated, AQP1 was suggested as an alternative pathway for oxygen diffusion. However, its caveolar localization is still debated. Membrane fractions from rabbit lung tissue homogenates have shown the presence of AQP1 (and Cav-1) in DRMs fractions (Palestini et al., 2003), and AQP1 colocalizes with Cav-1 in rat lung (Hill et al., 2005). Reports also suggested that AQP1 is in erythrocytes lipid rafts (Rungaldier et al., 2013) and caveolae extracts (Hill et al., 2005). However, western blot and proteomic analysis of caveolar and non-caveolar membrane domains isolated from 3T3-L1 cells identified AQP1 to be localized mainly in non-caveolar microdomains (Yao et al., 2009).

Other proteins localized in caveolae may present direct or indirect oxygen diffusion-associated functions. Indeed, caveolae are associated with proteins regulating an extensive range of functions, from trafficking to signaling (Head et al., 2014). Endothelial cell caveolae, involved in oxygen diffusion in alveolar capillary, exhibit diverse transmembrane proteins such as RTK, G-protein-coupled receptors, transforming growth factor receptors, etc. (Sowa, 2012). Ion channels have also been shown to be localized in caveolae. Many of these receptors are linked to metabolic functions with endpoint cells that are critical in regulating oxygen usage in these cells. Such is the case of cardiomyocytes a β-adrenergic receptor-mediated positive chronotropic (i.e., heart rate) and inotropic (i.e., contraction force) effect. Interestingly, evidence suggests caveolar localization of the β-adrenergic receptor in cardiomyocyte (Rybin et al., 2000; Steinberg, 2004), linking caveolar function as oxygen modulator with caveolae-localized receptors directly associated with cellular functions related to oxygen consumption. Caveolae localized TRPC function is regulated by cholesterol that leads to their localization in cholesterol-rich domains, assisted by the presence of CRAC motifs, at least in TRPV1 channels (Morales-Lazaro and Rosenbaum, 2017). It has been shown that caveolae can traduce mechanic stimulation (caveolae deformation) into caveolae-associated protein function. Membrane mechanical effect of osmotic stress induces Gα_q_/PLCβ-mediated Ca^2+^ currents in rat aortic smooth muscle (Guo et al., 2015; Yang and Scarlata, 2017). These observations suggest that caveolae-associated protein function may be subjected to membrane/caveolae mechanical condition, supporting the idea of caveolae serving as a mechanical sensor that regulate oxygen diffusion associated proteins.

## Cholesterol and Caveolae Adaptions to Hypoxia

### Membrane Cholesterol Adaptions Under Low Oxygen Availability

Diverse conditions, geographical (i.e., hypobaric hypoxia) and pathologically derived, result in low oxygen availability. Pioneering studies showed a correlation between cholesterol and oxygen transport (Buchwald et al., 2000b). Interestingly, several studies point to cholesterol-related adaptions in response to hypoxia, from sterol metabolism to membrane organization in cholesterol-rich domains. The acute exposure to hypobaric hypoxia induces modifications in gene expression. Ten hour exposure of rats to an environment that mimics 4600 m altitude (∼12.3% oxygen) resulted in downregulation of hepatic gene expression of sterol metabolism enzymes (3-hydroxy-3-methylglutaryl-CoA-synthase 1, farnesyl-diphosphate farnesyltransferase, squalene epoxidase, sterol-C4- methyl oxidase-like and 3β-hydroxysteroid dehydrogenase/Δ^5^-Δ^4^-isomerase) and SREBP1, with no significant reduction in plasma cholesterol (Dolt et al., 2007). Additionally, modifications in SREBP expression in response to hypoxia have been observed, leading to significant cellular and systemic effects. Key transcriptional regulators that respond to changes in PO_2_ such as HIF1 are associated with changes in cellular cholesterol content by regulating its uptake, which is critical in cancer cell physiology (Sundelin et al., 2012; Koizume and Miyagi, 2016).

Cholesterol adaption to hypobaric hypoxia constitutes a critical feature that relates sterols and oxygen availability and that have been well conserved during evolution. Considering that cholesterol may impose barriers to oxygen diffusion, subtle changes in its concentration in plasma membranes may facilitate the incorporation of oxygen from the environment so as optimize the distribution throughout the body. Human subjects exposed to high altitude present with variable cholesterol levels; prolonged exposure of human subjects to hypobaric hypoxia reduces plasma cholesterol (Ferezou et al., 1988). Individuals residing at high altitude (3,105 m∼14% oxygen) show higher plasmatic cholesterol (Temte, 1996). However, volunteers exposed for the first time to high altitudes (3,550 m∼13.2% oxygen) for 8 months revealed increased hematocrit and no significant changes in total plasma cholesterol but a significant increase in triglycerides (Siques et al., 2007). Homeostatic mechanisms induced by hypobaric hypoxia can be traced back to the cellular level at the air/blood barrier, namely alveolar endothelial Type II cells, basal membrane, and capillary endothelial cells. Rabbits exposed to 3 and 5 h of hypoxia (12% oxygen) developed an increase in phosphatidylethanolamine and cholesterol content in lung tissue cell membranes, with reduced Cav1 in DRMs (Botto et al., 2006). Hypoxia (5% oxygen) experiments performed in lung epithelial cells showed reduced Cav1 and cholesterol content in DRMs with a shift in Cav1 immunostaining from the membrane to intracellular localization (Botto et al., 2008). However, pulmonary artery endothelial cells from rats exposed chronically to hypobaric hypoxia (380 Torr∼10% oxygen) present a reduction in inward Ca^2+^ currents due to a reduction in membrane cholesterol (Paffett et al., 2011), suggesting that pulmonary artery endothelial cells physiology is susceptible to modification in response to hypoxia. Functional assays measuring Ca^2+^ inward currents in pulmonary artery endothelial cells have shown a reduction in cholesterol-controlled T-type voltage-gated Ca^2+^ channels and calcium release-activated calcium channel protein 1 Ca^2+^ entry due to the hypoxia-induced membrane cholesterol reduction (Zhang et al., 2017; Zhang B. et al., 2018). Notwithstanding, in the context of oxygen diffusion in alveolar air/blood barrier, the aforementioned membrane adaptions during hypoxia constitute a paradox: cell membrane cholesterol reduction may, from one side, facilitate oxygen diffusion in conditions in which the environmental oxygen supply is limited, however, on the other hand, hypoxia-induced cholesterol reduction disturbs functional organization of plasma membrane by means of Ca^2+^ influx, membrane potential, caveolae structural organization and potentially its role as a mechanosensor. In this regard, what in simple inspection may be a facilitating adaption to transfer more oxygen in conditions in which PO_2_ is low results ultimately in creating cellular damage.

The PO_2_ gradient is the driving force that allows gas diffusion from red blood cells to tissues with lower oxygen concentration. Membrane cholesterol modulation in peripheral tissues (i.e., transient reductions in membrane cholesterol) may represent a strategy that facilitates oxygen delivery. However, tissue hypoxia is an entirely different scenario with regulatory mechanisms that involve modifications in cholesterol synthesis by HIF-1α and 2α. HIF2α activation during hypoxia has been associated with fatty acid biology (Qu et al., 2011); pharmacological inhibition of HIF2α resulted in weight loss and fatty liver amelioration in obese mice (Feng et al., 2018). The increased hepatic activity of HIF2α by transient suppression of the *Vhl*^Δ IE^ induced an increase in hepatic and circulating cholesterol by repression of CYP7A1 (Ramakrishnan et al., 2014). Brain hypoxia-ischemia in neonatal mice increased cortex HMGCR and CYP46A1 after 24 h (Lu et al., 2018), suggesting that brain cholesterol metabolism adaption in response to hypoxia. Interestingly, epigenetic studies performed in Andean highlanders suggest that high altitude hypobaric hypoxia exposure affected HIF2α methylation (Childebayeva et al., 2019), opening the possibility to HIF2α activation regulation and ultimately, cholesterol. Nevertheless, how HIF2α-dependent fatty acid and cholesterol modifications affect plasma membrane organization, caveolae structure, and its potential role as an oxygen diffusion sensor in several tissues remains an open question. It has been shown that HIF2α activation leads to an increase in Cav1 expression in the *Vhl*^Δ IE^ mouse colon epithelial cells, leading to a decrease in occludin expression with a concomitant increase in colon barrier permeability (Xie et al., 2014).

One of the characteristic features in cancer cells is the abnormal growth rate and metabolic adaptions (Warburg effect). HIF cholesterol and hypoxia are crucial for cancer cell physiology (Koizume and Miyagi, 2016); it has been observed in prostate (Fredericks et al., 2013), and other forms of cancer (Kuzu et al., 2016; Ribas et al., 2016) increased cholesterol synthesis. Despite the altered cellular physiology features in cancer cells, this observation may suggest a role for cholesterol and oxygen availability within the cell to modulate growth and migration. BAECs enriched with cholesterol occupy a higher area compared with cholesterol-depleted cells (Hong et al., 2013). Early reports suggested the involvement of cholesterol and cell growth. Ascites tumor cells subjected to low-supplemented culture media showed low proliferation rates that were overcome by cholesterol supplementation (Haeffner et al., 1984). However, other reports point to an inverse relationship between cell size and membrane cholesterol content. Isolated rat subcutaneous adipocytes subpopulations showed that larger adipocytes had lower cholesterol concentration (Le Lay et al., 2001). Therefore, several questions can be addressed in this regard. Cell growth is an energy-demanding process that requires increased oxygen availability and consumption via mitochondrial activity, how is this compatible with the positive correlation between cell growth and cholesterol? How does transmembrane and/or aquaporin-mediated oxygen diffusion regulate under these circumstances?

### Caveolae-Dependent Tissue Specific Oxygen Distribution and Caveolae Role as an Oxygen Sensor

**FIGURE 4:**
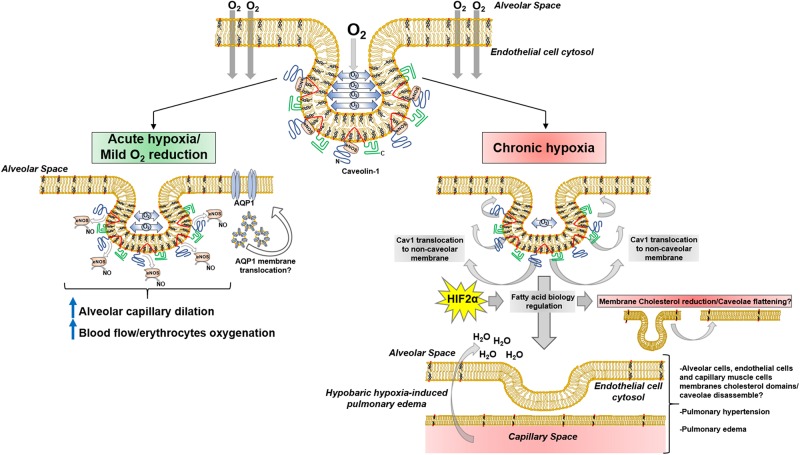
Lung Cav1/caveolae adaptions during hypoxia.

During the last 30 years, a high level of structural and functional complexity of lipid membranes has been described, where lipid rafts and caveolae develop a pivotal role in a pleiad of phenomena. A growing body of evidence suggests caveolae are partially involved in hypoxia-related mechanisms; high altitude hypobaric hypoxia has been thoroughly investigated because of oxygen-deprivation conditions like pulmonary edema. Pulmonary arterial smooth muscle and endothelial cells K^+^ and Ca^2+^ currents are altered during hypoxia (Archer et al., 2001, 2004). Inward Ca^2+^ currents mediated by TRPCs), has been suggested to play a key role in Ca^2+^ entry in lung artery muscle cells during hypoxia (Murray et al., 2006). Interestingly, K^+^ (i.e., K_v_1.5) and Ca^2+^ (i.e., TRPC1) channels are located in cholesterol-rich membrane domains and caveolae (Martens et al., 2001; Patel et al., 2005) in pulmonary artery muscle cells, thus playing a pivotal role in pulmonary physiology. Additionally, Cav-1 is linked to hypoxia-derived pulmonary hypertension (Mathew, 2014; Gilbert et al., 2016; Gao et al., 2017) and vascular remodeling mediated by PY-STAT3. Recent data suggest sustained PY-STAT3 activation in rats and neonatal calves exposed to chronic hypobaric hypoxia, showed no reduction in lung tissue and endothelial Cav-1 expression (Huang J. et al., 2019). This result opens the possibility of Cav-1 delocalization from caveolae, agreeing with previous data showing Cav-1 reduction in lung tissue membrane extracts and endothelial cells DRMs induced by hypoxia (Botto et al., 2006, 2008). In this regard, it is possible that a sequence of events occurs within caveolae after hypobaric hypoxia, leading to particular pathological scenarios in lung physiology. Subtle or initial reduction in oxygen diffusion may induce short-term response mechanisms by Cav-1/caveolae mechanosensor function, like eNOS modulation and lung capillary dilation; the persistence of hypobaric hypoxia may conduct Cav-1 delocalization from caveolae, co-adjuvated by HIF2α-mediated cholesterol reduction, and subsequently reductions of its expression, leading to pathological scenarios like pulmonary hypertension and hypobaric hypoxia-induced pulmonary edema (Figure 4).

## Open Questions and Concluding Remarks

With the accumulation of higher PO_2_ in the terrestrial atmosphere, sterols association with lipid membranes of organisms to limit oxygen diffusion represented a suitable evolutionary strategy, especially with the strong association between oxygen and cholesterol metabolism. Oxygen diffusion through lipid membranes has been thoroughly studied. Lipid membrane composition and complexity directly affect oxygen diffusion efficiency, impacting the acquisition of oxygen from the environment. This ultimately is critical to transport and delivery in peripheral tissues. Complex response mechanisms have been developed during evolution to ensure appropriate oxygen supply. In this regard, membrane structural specialization in functional domains enriched in cholesterol, like caveolae, has been observed to play a critical role as oxygen sensors. Hypobaric hypoxia constitutes a challenge that requires biophysical and biochemical strategies that will ensure oxygen diffusion. Interestingly, caveolae behave as a functional hub associated with hypobaric hypoxia response mechanisms. However, many questions are open regarding membranes and their functional (caveolae) domains in the oxygen diffusion process (i.e., How does oxygen diffusion effect in lipid membrane order and protein function; does caveolar mechanotransduction function to detect local oscillations in oxygen diffusion in lung epithelial and tissues submitted to hypoxia, what role do caveolae play in hypoxia during aging; if cholesterol is a crucial determinant for oxygen diffusion, how does tissue-specific cell membranes cholesterol concentration affects oxygen supply; how does cholesterol metabolism impairment (i.e., during aging) affect tissue oxygen delivery/diffusion in normal and hypoxic conditions; what other caveolin-associated proteins may behave as oxygen sensors). In this regard, it can be observed that an intimate relationship between caveolae and lung physiology exists. The selection of caveolae as functional platforms in pulmonary tissues, its association with hypoxia-related response mechanisms, also associated with cholesterol biosynthesis, and the structural role in membranes suggests implications for oxygen transport that interact with pressures to maintain physiology and manage pathophysiology in living systems.

## Author Contributions

All authors listed have made a substantial, direct and intellectual contribution to the work, and approved it for publication.

## Conflict of Interest

HP has equity as a founder in CavoGene Life Sciences Holdings, LLC. The remaining author declares that the research was conducted in the absence of any commercial or financial relationships that could be construed as a potential conflict of interest.

## Abbreviations

ABCA3: ATP-binding cassette A3 transporter
ABCG1: ATP-binding cassette G1 transporter
AQP1: aquaporin 1
BAECS: bovine aorta endothelial cells
CARC: inverted CRAC domain
Cav1-3: caveolins 1 to 3
CRAC: cholesterol recognition/interaction amino acid consensus sequences
CYP7A1: cholesterol hydroxylase 7 α-hydroxylase
DMO_2_: membrane conductance to oxygen
DMPC: 1,2-dimyristoyl-*sn*-glycero-3-phosphocholine
DOPC: 1,2-dioleoyl-sn-glycero-3-phosphocholine
DPPC: 1,2-dipalmitoyl-sn-glycero-3-phosphocholine
DRMs: detergent-resistant membranes
eNOS: endothelial nitric oxide synthase
EPR: electron paramagnetic resonance spectroscopy
ERK1/2: extracellular signal-regulated kinase 1/2
G α_q_/PLC β: G α_q_ phospholipase C β
GOE: Great Oxygenation Event
HIF: hypoxia-inducible factor
HIF-1 α: hypoxia-inducible factor 1 α
HMGCR: 3-hydroxy-3-methylglutaryl-CoA-reductase
M β CD: methyl- β-cyclodextrin
MLEC: murine lung endothelial cells
MPP1: membrane palmitoylated protein 1
NOS: nitric oxide synthase
Nrf2: nuclear factor erythroid 2-related factor 2
PC: phosphatidylcholine
PECAM1: platelet endothelial cell adhesion protein 1
PO_2_: oxygen pressure
POPC: 1-palmitoyl-2-oleoyl-sn-glycero-3-phosphocholine
POPE: 1-palmitoyl-2-oleoyl-*sn*-glycero-3-phosphocholine
PY-STAT3: tyrosine-phosphorylated signal transducer and activator of transcription factor
ROS: reactive oxygen species
RTK: tyrosine kinase receptor
SP-C: surfactant peptide C
SREBP1: sterol regulatory element binding protein 1
Tm: melting temperature
TRPC: transient receptor potential channels
TRPCs: canonical transient receptor potential channels
*Vhl*^Δ IE^: Von Hippel-Lindau factor.
